# A cubic algorithm for the generalized rank median of three genomes

**DOI:** 10.1186/s13015-019-0150-y

**Published:** 2019-07-26

**Authors:** Leonid Chindelevitch, Sean La, Joao Meidanis

**Affiliations:** 1 School of Computing Science, Simon Fraser University, Burnaby, Canada; 2 Institute of Computing, University of Campinas, Campinas, Brazil

**Keywords:** Comparative genomics, Ancestral genome reconstruction, Phylogenetics, Rank distance

## Abstract

**Background:**

The area of genome rearrangements has given rise to a number of interesting biological, mathematical and algorithmic problems. Among these, one of the most intractable ones has been that of finding the median of three genomes, a special case of the ancestral reconstruction problem. In this work we re-examine our recently proposed way of measuring genome rearrangement distance, namely, the rank distance between the matrix representations of the corresponding genomes, and show that the median of three genomes can be computed exactly in polynomial time $O(n^\omega )$, where $\omega \le 3$, with respect to this distance, when the median is allowed to be an arbitrary orthogonal matrix.

**Results:**

We define the five fundamental subspaces depending on three input genomes, and use their properties to show that a particular action on each of these subspaces produces a median. In the process we introduce the notion of *M*-stable subspaces. We also show that the median found by our algorithm is always orthogonal, symmetric, and conserves any adjacencies or telomeres present in at least 2 out of 3 input genomes.

**Conclusions:**

We test our method on both simulated and real data. We find that the majority of the realistic inputs result in genomic outputs, and for those that do not, our two heuristics perform well in terms of reconstructing a genomic matrix attaining a score close to the lower bound, while running in a reasonable amount of time. We conclude that the rank distance is not only theoretically intriguing, but also practically useful for median-finding, and potentially ancestral genome reconstruction.

## Background

The genome median problem consists of computing a genome *M* that minimizes the sum $d(A, M) + d(B, M) + d(C, M)$, where *A*, *B*, and *C* are three given genomes and $d(\cdot ,\cdot )$ is a distance metric that measures how far apart two genomes are, and is commonly chosen to correlate with evolutionary time. In this paper, we present a polynomial-time algorithm for the computation of a median for the rank distance. We call it a generalized median because, despite attaining a lower bound on the best score with respect to the rank distance, it may not be a genome in all cases. However, we report on experiments that show that the median is genomic in the majority of the cases we examined, including real genomes and artificial genomes created by simulation, and when it is not, a genome close to the median can be found via an efficient post-processing heuristic.

This result is a significant improvement on the first algorithm for generalized medians with respect to the rank distance [1], which makes it fast enough to be used on real genomes, with thousands of genes. Our experiments deal with genomes with up to 1000 genes, but the measured running times of the algorithm and their extrapolation suggest that reaching tens of thousands of genes is feasible.

Our work builds upon a recent result from our group that shows the first polynomial-time algorithm for rank medians of orthogonal matrices [1], delivering an alternative specific to genomes which avoids any floating-point convergence issues, guarantees the desirable properties of symmetry and majority adjacency/telomere conservation, and provides a speed-up from ${\varTheta }(n^{1 + \omega })$ to ${\varTheta }(n^ \omega )$ in the worst case, where $\omega$ is the exponent of matrix multiplication known to be less than 2.38 [2], but close to 3 on practical instances. Prior to this result, there were fast, polynomial-time median algorithms for simpler distances, such as the breakpoint distance [3] and the SCJ distance [4]. In contrast, for more sophisticated distances such as the inversion distance [5] and the DCJ distance [3], the median problem is NP-hard, meaning that it is very unlikely that fast algorithms for it exist. The rank distance is equal to twice the algebraic distance [6], which in turn is very close to the widely used DCJ distance [7]. More specifically, it assigns a weight of 1 to cuts and joins, and a weight of 2 to double swaps; it is known that the rank distance equals the total weight of the smallest sequence of operations transforming one genome into another under this weighting scheme [8]. Therefore, it is fair to place the rank distance among the more sophisticated distances, that take into account rearrangements such as inversions, translocations, and transpositions, with weights that correlate with their relative frequency.

A more complete distance will also take into account content-changing events, such as duplications, gene gain and loss, etc. We hope that our contribution provides significant insight towards studies of more complex genome distances.

### Definitions

Let $n \in {\mathbb {N}}$ be an integer and let ${\mathbb {R}}^{n \times n}$ be the set of $n \times n$ matrices with entries in ${\mathbb {R}}$. Following [6], we say that a matrix *M* is *genomic* when it is:

- *Binary*, i.e. $$M_{ij} \in \{0,1\} \  \forall \  i,j$$
- *Orthogonal*, i.e. $M^{T} = M^{-1}$ (so the columns of *M* are pairwise orthogonal)
- *Symmetric*, i.e. $$M^{T} = M$$ (so $$M_{ij} = M_{ji} \  \forall \  i,j$$).

Strictly speaking, *n* must be even for a genomic matrix, because *n* is the number of gene extremities, and each gene contributes two extremities, its head and its tail [6]. However, most of our results apply equally well to all integers *n*.

A genomic matrix *M* defines a permutation $\pi$ via the relationship

$$\begin{aligned} \pi (i) = j \iff M_{i,j} = 1. \end{aligned}$$

It is easy to see that the permutation $\pi$ corresponding to a genomic matrix is a product of disjoint cycles of length 1 and 2. The cycles of length 1 correspond to *telomeres* while the cycles of length 2 correspond to *adjacencies*. The correspondence between a genome *G* and a genomic matrix *M* is defined by

$$\begin{aligned}  M_{i,j}  = 1  \iff & i \ne j \text{ and } (i,j) \  \text{ is } \text{ an } \text{ adjacency } \text{ in } \  G, \  \text{ or } \\  & i = j \  \text{ and } \  i \  \text{ is } \text{ a } \text{ telomere } \text{ in } \  G. \end{aligned}$$

### Rank distance

The rank distance $d(\cdot , \cdot )$ [9] is defined on ${\mathbb {R}}^{n \times n}$ via

$$\begin{aligned} d(A,B) = r(A-B), \end{aligned}$$

where *r*(*X*) is the *rank* of the matrix *X*, defined as the dimension of the *image* (or column space) of *X* and denoted ${{\,\text{im}\,}}(X)$. This distance is a metric and is equivalent to the Cayley distance between the corresponding permutations when *A* and *B* are both permutation matrices [1, 6].

The relevance of the rank distance for genome comparison stems from the fact that some of the most frequent genome rearrangements occurring in genome evolution, such as inversions, transpositions, translocations, fissions and fusions, correspond to a perturbation of a very low rank (between 1 and 4, depending on the operation) of the starting genomic matrix. This suggests that the rank distance may be a good indicator of the amount of evolution that separates two genomic matrices. We previously reviewed its relationship to other distances [1].

### The median problem and invariants

Given three matrices *A*, *B*, *C*, the median *M* is defined as a global minimizer of the *score* function $d(M;A,B,C) := d(A,M) + d(B,M) + d(C,M)$.

In previous work we identified three important invariants for the median-of-three problem. The first invariant is defined as:

$$\begin{aligned}  \beta (A,B,C) := \frac{1}{2} [d(A,B) + d(B,C) + d(C,A)]. \end{aligned}$$

This invariant is known to be integral if *A*, *B*, and *C* are orthogonal matrices, which include genomic matrices and permutation matrices as special cases [1].

The first invariant is also a lower bound for the score: $d(M;A,B,C) \ge \beta (A,B,C)$, with equality if and only if

1 $$\begin{aligned} & d(X,M) + d(M,Y) \nonumber \\  & \quad = d(X,Y) \  \text {for any distinct} \  X, Y   \in \{A,B,C\}. \end{aligned}$$

The second invariant is the dimension of the “triple agreement” subspace [1]:

2 $$\begin{aligned}  & \alpha (A,B,C) := \dim (V_1), \  {\text{where}} \\  & V_1 := \{x \in {\mathbb {R}}^{n}|Ax = Bx = Cx\}. \end{aligned}$$

Finally, the third invariant combines the first two with the dimension *n*:

3 $$\begin{aligned}  \delta (A,B,C)  := \alpha (A,B,C) + \beta (A,B,C) - n. \end{aligned}$$

This invariant is known to be non-negative if *A*, *B*, and *C* are orthogonal [1]. We therefore call it the *deficiency* of *A*, *B* and *C*, by analogy with the deficiency of a chemical reaction network defined in the work of Horn, Jackson and Feinberg [10]. We recall here our “deficiency zero theorem” for medians of permutations [1].

#### **Theorem 1**

(Deficiency Zero Theorem) *Let* *A*, *B*, *C* *be permutations with* $\delta (A,B,C) = 0$. *Then the median is unique, and can be found in* $O(n^2)$ *time*.

### The five subspaces and their dimensions

The inputs of a median-of-three problem partition ${\mathbb {R}}^n$ into five subspaces [6], which we describe in this section.

The “triple agreement” subspace $V_1 = V(.A.B.C.)$ is defined in Eq. (2), and is the subspace of all vectors on which all three matrices agree. Its dimension is $\alpha (A,B,C)$, by definition.

The subspace $V_2 := V(.AB.C.) \cap V_1^{\perp }$ is defined via $V_1$ and the subspace

$$\begin{aligned} V(.AB.C) := \{x \in {\mathbb {R}}^n|Ax = Bx\}. \end{aligned}$$

The dimension of *V*(.*AB*.*C*) is precisely $c(\rho ^{-1} \sigma )$, where $\rho$ and $\sigma$ are the permutations corresponding to *A* and *B*, respectively, and $c(\pi )$ is the number of cycles (including fixed points) in a permutation $\pi$. This follows from this observation:

4 $$\begin{aligned} & Ax = Bx \iff A^{-1}Bx = x \nonumber \\  & \quad \iff x \  \text {is constant on every cycle of}  \rho ^{-1} \sigma . \end{aligned}$$

Since $V_1 \subseteq V(.AB.C)$, it follows that a basis of $V_1$ can be extended to a basis of *V*(.*AB*.*C*) with vectors orthogonal to those spanning $V_1$, so that

$$\begin{aligned} \dim (V_2)=  &  {} \dim (V(.AB.C.) \cap V_1^{\perp }) \\=  &  {} \dim (V(.AB.C.) - \dim (V_1) \\=  &  {} c(\rho ^{-1} \sigma ) - \alpha . \end{aligned}$$

We can apply a similar reasoning to the subspaces $V_3 := V(.A.BC.) \cap V_1^{\perp }$ and $V_4 := V(.AC.B) \cap V_1^{\perp }$, where $V(.A.BC.) := \{x \in {\mathbb {R}}^n|Bx = Cx\}$ and $V(.AC.B) := \{x \in {\mathbb {R}}^n|Cx = Ax\}$, to get

$$\begin{aligned} \dim (V_2) & =   c(\rho ^{-1} \sigma ) - \alpha ; \\ \dim (V_3) & =  c(\sigma ^{-1} \tau ) - \alpha ; \\ \dim (V_4) & =  c(\tau ^{-1} \rho ) - \alpha , \end{aligned}$$

where $\tau$ is the permutation corresponding to *C*. We call the spaces $V_2, V_3, V_4$ the “pairwise agreement” subspaces because they contain vectors on which two, but not all three, of the input matrices agree.

It was shown by Zanetti et al. [6] that

5 $$\begin{aligned} {\mathbb {R}}^n = V_1 \oplus V_2 \oplus V_3 \oplus V_4 \oplus V_5, \end{aligned}$$

where $V_5$ is the subspace orthogonal to the sum of the four “agreement” subspaces (hence called the “disagreement” subspace), and the $\oplus$ notation represents a direct sum, i.e. $V_i \cap V_j = \{0\}$ whenever $1 \le i < j \le 5$. For each $1 \le j \le 5$, we also define the projector $P_j$, as the projector onto $V_j$ along $\oplus _{i \ne j} V_i$. After that Eq. (5) can also be equivalently written as $\sum _{j = 1}^{5} P_j = I$.

Since $V_5$ is the last term in the direct sum decomposition of ${\mathbb {R}}^n$, we get that

$$\begin{aligned} \dim (V_5) & = n - \sum _{i=1}^{4} \dim (V_i) \\  & = n + 2 \alpha - (c(\rho ^{-1} \sigma ) + c(\sigma ^{-1} \tau ) + c(\tau ^{-1} \rho )) \\  & = n + 2 \alpha (A,B,C) - (3n - 2\beta (A,B,C)) \\  & = 2 (\alpha + \beta \ - n) = 2 \delta (A,B,C). \end{aligned}$$

### A specific example

Let us now look at a specific example (which is one of our simulated inputs). Let

$$\begin{aligned} & A = (24)(39)(68)(10 \  11), \\  & B = (27)(38)(45)(69)(10 \  11), \\  & C = (23)(45)(67)(89)(10 \  11). \end{aligned}$$

We use $n = 12$ although 12 is a singleton in all inputs. First note that $AB = (2745)(36)(89)$, $BC = (286)(379)$, and $CA = (25438769)$, so $\alpha (A,B,C) = 5$ because the triple agreement space is spanned by the indicator vectors of the sets $\{1\}, \{2,3,4,5,6,7,8,9\}, \{10\}, \{11\}, \{12\}$. Furthermore, by counting the cycles in the products above we get $d(A,B) = 5, d(B,C) = 4, d(C,A) = 7$, so $\beta (A,B,C) = 8$ and $\delta (A,B,C) = 1$. The dimensions of the subspaces $V_1$ through $V_5$ are thus 5, 2, 3, 0, and 2.

We note that we can ignore the common telomeres 1 and 12 as well as the common adjacency $(10 \  11)$ because we can assume they will be present in a median (see Theorem 1 in [6]). Thus, we can simplify our example by adding the known adjacencies and telomeres to the median and removing them from the input. After renumbering the remaining extremities from 1 to 8, the input becomes

$$\begin{aligned} A^{\prime}  & = (13)(28)(57), \, B^{\prime} = (16)(27)(34)(58), \\ C^{\prime}  & = (12)(34)(56)(78). \end{aligned}$$

Now the invariants reduce to $\alpha (A',B',C')=1$, $\beta (A',B',C')=8$, $\delta (A',B',C')=1$, and the subspace dimensions become 1, 2, 3, 0, and 2, respectively.

### Highlights for small *n*

To gain insight into the median problem, we scrutinized the problem of computing the median for all genomic matrices for $n = 3$ to $n = 8$. For each *n*, we classified the input matrices in a number of equivalent cases. For $n = 3$ and $n = 4$, we computed all the medians for all cases. For $n = 5$ and higher, we concentrated on the cases with positive deficiency $\delta$, given that cases with $\delta = 0$ are easy (Theorem 1). We tested an algorithm, which we call algorithm ${{\mathcal {A}}}$, that is a modification of the algorithm in [6] where *M* agrees with the corresponding input on the 4 “agreement subspaces”, but mimics the identity matrix on the subspace $V_5$. More specifically, Algorithm ${{\mathcal {A}}}$, given genomic matrices *A*, *B*, and *C*, returns matrix $M_I$ defined as follows:

$$\begin{aligned} M_I(v) = \left\{ \begin{array}{ll} Av  & \quad \text{ if } \, v \in V_1 \\ Av  & \quad \text{ if } \, v \in V_2 \\ Bv  & \quad \text{ if } \, v \in V_3 \\ Cv  & \quad \text{ if } \, v \in V_4 \\ v  & \quad \text{ if } \, v \in V_5 \\ \end{array}\right. \end{aligned}$$

where the subspaces $V_1, \ldots , V_5$ were defined in the section “The five subspaces and their dimensions”.

We observed that in all cases we examined the result $M_I$ was an orthogonal matrix, and algorithm ${\mathcal {A}}$ was able to find a median attaining the lower bound $\beta (A,B,C)$; we prove both of these facts in the remainder of this paper.

In the Appendix, we provide two detailed examples of some of the situations that can arise when trying to compute all the medians. The first one demonstrates that in some cases, all the medians form a group under multiplication; this situation can only occur when the identity is a median, and seems to arise due to certain symmetries among the inputs. The second one demonstrates that medians do not have to be orthogonal, by providing three genomic matrices of size $n = 5$ which admit a family of non-orthogonal medians.

## $M_I$ and its computation

Following our experiments with algorithm ${\mathcal {A}}$, we conjectured—and proved—that it always produces a median when the inputs are genomic matrices. Furthermore, we proved that this median is always orthogonal, symmetric, and has rows and columns that add up to 1. It also contains only rational entries, and in our experiments, these entries are 0 and 1 most of the time, meaning that the median produced by algorithm ${\mathcal {A}}$ is actually genomic. For the few cases when this property does not hold, we introduce two heuristics in the next section.

The rest of this section is organized as follows: we begin by defining $M_I$, the output of algorithm ${\mathcal {A}}$, and provide sufficient conditions for its optimality in the “Definition of M_I_ and sufficient conditions for optimality” section. We prove its symmetry in the “Symmetry of M_I_” section and its orthogonality in the “Orthogonality of M_I_” section. We sketch the proof of its optimality in the “Optimality of M_I_” section, providing the complete version in the Appendix. We prove a result showing that $M_I$ contains any adjacencies and telomeres common to at least two of the three input genomes in the “Conservation of common adjacencies and telomeres” section. Lastly, we discuss how to compute $M_I$ efficiently in the “Computation of M_I_” section.

### Definition of $M_I$ and sufficient conditions for optimality

We start with a general result on matrices that mimic the majority of inputs in $V_1$ through $V_4$, and mimic a certain matrix *Z* in $V_5$.

#### **Definition 1**

Let *A*, *B*, *C* be permutation matrices of size *n*, and let *Z* be a fixed matrix of size *n*. As above, let $V_1$ through $V_5$ be the 5 subspaces in the direct sum decomposition of ${\mathbb {R}}^n$ induced by *A*, *B*, *C*, and let $P_j$ be the projector onto $V_j$ for $1 \le j \le 5$. We define $M_Z := AP_1 + AP_2 + BP_3 + CP_4 + ZP_5$ as the matrix that agrees with the corresponding inputs on the “agreement spaces” $V_1, V_2, V_3, V_4$ and acts by the operator *Z* on the “disagreement space” $V_5$.

#### **Definition 2**

Let *A*, *B*, *C* be permutation matrices, and let *Z* be a fixed matrix, and let $V_1$ through $V_5$ be the 5 subspaces in the direct sum decomposition of ${\mathbb {R}}^n$ induced by *A*, *B*, *C*. We define $V_Z^{A} := \{x + y | x \in V_3, y \in V_5, A(x+y) = Bx+Zy\}$, and similarly, $V_Z^{B} := \{x + y | x \in V_4, y \in V_5, B(x+y) = Cx+Zy\}$ and $V_Z^{C} := \{x + y | x \in V_2, y \in V_5, C(x+y) = Ax+Zy\}.$

#### **Lemma 1**

*Let* $M_Z$ *be the matrix in Definition* 1 *and let* $V_Z^A$, $V_Z^B$, $V_Z^C$ *be the subspaces in Definition* 2. *Then the score of* $M_Z$ *with respect to* *A*, *B*, *C* *is* $s(M_Z) := \beta (A,B,C) + 3 \delta (A,B,C) - (\dim (V_Z^{A}) + \dim (V_Z^{B}) + \dim (V_Z^{C})).$

#### *Proof*

Recall Eq. (5): ${\mathbb {R}}^n = \bigoplus _{i=1}^{5} V_i$. By construction, $M_Z$ agrees with *A* on the subspaces $V_1, V_2, V_4$ so those do not contribute to the rank of $M_Z-A$. Therefore, by the rank plus nullity theorem,

$$\begin{aligned} d(M_Z,A)=  &  {} \dim (V_3) + \dim (V_5) \\  & - \dim \{z \in V_3 + V_5| Az = M_Z z\}. \end{aligned}$$

However, the space whose dimension is subtracted can also be rewritten as

$$\begin{aligned} \{z = x + y | x \in V_3, y \in V_5, A(x+y) = Bx + Zy\} =: V_Z^{A}, \end{aligned}$$

since $M_Z$ acts by *B* on $V_3$ and by *Z* on $V_5$, by Definition 1. We combine this result with similar results for *B* and *C* to deduce that

6 $$\begin{aligned} d(M_Z,A) & = \dim (V_3) + \dim (V_5)  - \dim (V_Z^{A}); \end{aligned}$$

7 $$\begin{aligned} d(M_Z,B) & = \dim (V_4) + \dim (V_5) - \dim (V_Z^{B}); \end{aligned}$$

8 $$\begin{aligned} d(M_Z,C) & = \dim (V_2) + \dim (V_5) - \dim (V_Z^{C}). \end{aligned}$$

By adding these up and using the fact that $\dim (V_5) = 2 \delta (A,B,C)$ and $\dim (V_2) + \dim (V_3) + \dim (V_4) = n - \dim (V_5) - \alpha (A,B,C)$ we obtain the desired conclusion. $\square$

#### **Lemma 2**

*The median candidate* $M_Z$ *from Lemma* 1 *attains the lower bound if and only if* $\dim (V_Z^{A}) = \dim (V_Z^{B}) = \dim (V_Z^{C}) = \delta (A,B,C)$.

#### *Proof*

We start by considering Eq. (6) in the proof of Lemma 1, since the other two are analogous. By the necessary conditions for optimality in Eq. (1),

9 $$\begin{aligned} d(M_Z,A) & =  \beta (A,B,C) - d(B,C) \nonumber \\ & =   \beta (A,B,C) - (n - c(\sigma ^{-1} \tau )). \end{aligned}$$

On the other hand, we have $\dim (V_3) = c(\sigma ^{-1} \tau ) - \alpha (A,B,C)$ and $\dim (V_5) = 2 \delta (A,B,C)$, so by combining Eq. (6) with Eq. (9) we obtain

$$\begin{aligned} \dim (V_Z^{A}) &=  \dim (V_3) + \dim (V_5)  - d(M_Z,A) \\& =  \beta (A,B,C) + \alpha (A,B,C) - n \\&=  \delta (A,B,C). \end{aligned}$$

For the sufficiency, it is enough to check that when all three spaces have this dimension, then $s(M_Z) = \beta (A,B,C)$, which follows immediately from Lemma 1. $\square$

### Symmetry of $M_I$

We first define a new term that we call an *M*-stable subspace; this is closely related to the notion of an *M*-invariant subspace [11], which is a subspace *V* such that $MV \subseteq V$, but with the additional specification that the dimensions are preserved. More specifically, we propose the following

#### **Definition 3**

Let *M* be an invertible $n \times n$ matrix and let *V* be a subspace of ${\mathbb {R}}^n$. Then *V* is an *M*-stable subspace if and only if $MV = V$.

We have the following properties that we prove in the Appendix:

#### **Theorem 2**

*Let* *M* *and* *N* *be invertible matrices. Then*

a.: *If* *V*, *W* *are two* *M*-*stable subspaces, then so are* $V \cap W$ *and* $V + W$.
b.: *If* *M* *is symmetric and* *V* *is an* *M*-*stable subspace*, *then so is* $V^{\perp }$.
c.: *If* $M^2 = I = N^2$ *then the subspace* $\{x|Mx = Nx\}$ *is* *M* *-stable and N-stable.*

We note that Part b. can be false if *M* is not symmetric; for instance, when $$M = \begin{pmatrix} 1  & {} 1\\ 0  & {} 2 \end{pmatrix}$$, we have the *M*-stable subspace spanned by $[1,1]^{T}$ whose orthogonal complement, spanned by $[1,-1]^{T}$, is not *M*-stable.

An easy but useful consequence of this theorem is the following

#### **Lemma 3**

*Let* *A*, *B*, *C* *be involutions. Then the subspace* $V_1$ *is* *A*-*stable*, *B*-*stable and* *C*-*stable; the subspace* $V_2$ *is* *A*-*stable and* *B*-*stable; the subspace* $V_3$ *is* *B*-*stable and* *C*-*stable; and the subspace* $V_4$ *is* *A*-*stable and* *C*-*stable*.

#### *Proof*

We begin by showing that $V_1$ is *A*-stable. Indeed, $V_1 = \{x|Ax = Bx = Cx\} = \{x|Ax = Bx\} \cap \{x|Ax = Cx\}$ is the intersection of two subspaces, each of which is *A*-stable by part c of Theorem 2, and therefore is itself *A*-stable by part a. The fact that it is also *B*-stable and *C*-stable follows by symmetry.

Similarly, $V_2 = \{x|Ax = Bx\} \cap V_1^{\perp }$ is the intersection of two subspaces that are *A*-stable by parts c and b of Theorem 2, respectively, and so is *A*-stable itself by part a. By symmetry, $V_2$ is also *B*-stable, and the same reasoning applied to $V_3$ and $V_4$ shows that they are stable for the two involutions defining them. $\square$

#### **Theorem 3**

$M_I$ *is always symmetric for involutions* *A*, *B* *and* *C*.

#### *Proof*

To prove the symmetry of an $n \times n$ matrix *M*, it is sufficient to show that

10 $$\begin{aligned} x^{T} M y = y^{T} M x \quad  \forall \  x,y \in {\mathbb {R}}^n. \end{aligned}$$

By linearity, it is enough to show this for a set of basis vectors of ${\mathbb {R}}^n$. We choose the basis of ${\mathbb {R}}^n$ to be the union of the bases for the subspaces $V_i$ for $i=1$ to $i=5$. Now Lemma 3 shows that for any of these subspaces, $x \in V_i$ implies $M_I x \in V_i$. Indeed, this is clear for $i=1$ to $i=4$, since the corresponding vector gets projected into its own subspace $V_i$ and then acted on by an involution that fixes $V_i$. This is also clear for $i=5$ since any vector in $V_5$ is fixed by $M_I$.

Suppose first that *x*, *y* are two vectors from different subspaces, say $x \in V_i, y \in V_j$, with $i < j$ without loss of generality; then we consider three cases:

Case A $i = 1$ and $j \in \{2, 3, 4, 5\}$; since $V_1$ and $V_j$ are mutually orthogonal, we have $x^{T} M_I y = 0 = y^{T} M_I x$, since $M_I x \in V_1$ and $M_I y \in V_j$ by the result above.
Case B $i \in \{2, 3, 4\}$ and $j = 5$; since $V_i$ and $V_5$ are mutually orthogonal, we have $x^{T} M_I y = 0 = y^{T} M_I x$, since $M_I x \in V_i$ and $M_I y \in V_5$ by the result above.
Case C $i \in \{2, 3\}$ and $j \in \{i+1, \ldots , 4\}$; we consider the case $i = 2$ and $j = 3$, as the others follow by symmetry. Since $M_I = B$ on both $V_2$ as well as $V_3$,
  $$\begin{aligned} x^{T} (M_I y) & =  x^{T} (B y) = x^{T} B^{T} y =  (B x)^{T} y = \langle Bx, y\rangle \\ & =  y^{T} (B x) = y^{T} (M_I x). \end{aligned}$$

Now, suppose that *x*, *y* are two vectors from the same subspace, say $x,y \in V_i$. In this case, the matrix $M_I$ acts on $V_i$ via a symmetric matrix, and the same argument as in the previous equation shows equality, proving the desired result. $\square$

### Orthogonality of $M_I$

#### **Theorem 4**

$M_I$ *is always orthogonal for involutions* *A*, *B*, *and* *C*.

The proof proceeds along very similar lines to the proof that $M_I$ is symmetric, and is provided in the Appendix.

### Optimality of $M_I$

To show the optimality of $M_I$, it suffices to show that $\dim (V_I^{C}) \ge \delta (A,B,C)$, since symmetry implies that the same holds for $\dim (V_I^{A})$ and $\dim (V_I^{B})$, and then Lemma 1 shows that $M_I$ is a median because it achieves the lower bound.

Recall that the definition of $V_I^{C}$ asks for vectors $x + y$ such that *x* is in $V_2$, *y* is in $V_5$, and $C(x + y) = Ax + y$, or $(C - A)x + (C - I)y = 0$. The main idea is to show that it is enough to restrict ourselves to vectors *x* such that $(A - I)x = 0$, meaning that the equation simply becomes $(C - I)(x+y) = 0$. The full details are provided in the Appendix.

### Conservation of common adjacencies and telomeres

We say that an adjacency *i*, *j* is *present* in a matrix *M* if $M_{ij} = 1 = M_{ji}$, $M_{kj} = 0 = M_{jk}$ for any $k \ne i$, and $M_{ik} = 0 = M_{ki}$ for any $k \ne j$. Similarly, we say that a telomere *i* is *present* in a matrix *M* if $M_{ii} = 1$ and $M_{ik} = 0 = M_{ki}$ for any $k \ne i$. In other words, the association of *i* to *j* (for an adjacency) or to *i* (for a telomere) is unambiguous according to *M*. We now show that any adjacencies or telomeres common to 2 of 3 input genomes are present in any orthogonal median of three genomes, including $M_I$.

#### **Theorem 5**

*Let* *A*, *B*, *C* *be three genomic matrices with median* *M*. *If* $A_{ij} = 1 = B_{ij}$ *for some* *i*, *j*, *then* $M_{ij} = 1 = M_{ji}$, $M_{kj} = 0 \  \forall \  k \ne i$, *and* $M_{ki} = 0 \  \forall \  k \ne j$.

#### *Proof*

By optimality of $M_I$ shown in the previous section, any median *M* of three genomes attains the lower bound $\beta (A,B,C)$ on the score. Hence, by Eq. (1) it must satisfy $d(A,M) + d(M,B) = d(A,B)$. By Corollary 1 in [1] it follows that for any vector *x* with $Ax = Bx$, we also have $Mx = Ax$. We have two cases:

Case A $i = j$; then, taking $x = e_i$, the *i*th standard basis vector, we get that $Ax = Bx = x$, so $Mx = x$ as well. It follows that the *i*th column of *M* is $e_i$, so that $M_{ij} = M_{ii} = M_{ji} = 1$ and $M_{kj} = M_{ki} = 0 \  \forall \  k \ne i$, as required.
Case B $i \ne j$; then taking $x = e_i + e_j$ and $y = e_i - e_j$, we get that $Ax = Bx = x$ and $Ay = By = -y$, so that $Mx = x$ and $My = -y$ as well. By linearity, we take the half-sum and half-difference of these equations to get $Me_i = e_j$ and $Me_j = e_i$. The first of these implies that $M_{ij} = 1$ and $M_{kj} = 0 \  \forall \  k \ne i$, while the second one implies that $M_{ji} = 1$ and $M_{ki} = 0 \  \forall \  k \ne j$, as required.

$$\square $$

#### **Corollary 1**

*If* *M* *is an orthogonal median of genomic matrices* *A*, *B*, *C*, *and* $A_{ij} = 1 = B_{ij}$ *for some pair* *i*, *j*, *then* $M_{jk} = 0 \  \forall \  k \ne i$. *In particular, any adjacency or telomere common to 2 out of 3 input genomes is present in* $M_I$.

#### *Proof*

The first statement follows immediately from Theorem 5 and orthogonality. The second statement is clear for telomeres, and follows for adjacencies since an adjacency *i*, *j* is common to *A* and *B* if and only if $A_{ij} = B_{ij} = 1 = B_{ji} = A_{ji}$. $\square$

### Computation of $M_I$

In order to compute $M_I$ we need the projection matrices $P_j$, which require a basis matrix $B_j$ for each of the spaces $V_j$, for $1 \le j \le 5$, as well as a nullspace matrix $N_j$ for $2 \le j \le 4$ [6]. However, it turns out that we can dispense with the nullspace matrices altogether and bypass the computation of $B_5$, which tends to be complicated, by using column-wise matrix concatenation $[\cdot ,\cdot ]$ and the following formula:

11 $$\begin{aligned} M_I =  I + ([AB_1, AB_2, BB_3, CB_4] - B_{14}) (B_{14}^{T} B_{14})^{-1} B_{14}^{T}, \end{aligned}$$

where $$B_{14} := [B_1, B_2, B_3, B_4]$$.

To verify this equation, it suffices to check that the right-hand side agrees with $M_I$ on the basis vectors of each subspace $V_j$, for $1 \le j \le 5$. This is clear for $V_5$ since $B_{14}^{T} x = 0 \  \forall \  x \in V_5$, and is also true for the basis vectors of $V_j$ for $1 \le j \le 4$ since Eq. (11) implies that $M_I B_{14} = [AB_1, AB_2, BB_3, CB_4]$.

It is easy to compute a basis $B_1$ for the triple agreement space $V_1$. Indeed, we note that, by Eq. (4),

$$\begin{aligned} x \in V_1\iff  &  {} Ax = Bx = Cx\\\iff  &  {} x \text { is constant on the cycles of } \rho ^{-1} \sigma \  \text {and} \  \sigma ^{-1} \tau , \end{aligned}$$

where $\rho , \sigma , \tau$ are the permutations corresponding to *A*, *B*, *C*, respectively. The computation of $\rho ^{-1} \sigma$ and $\sigma ^{-1} \tau$ takes *O*(*n*) time, and $V_1$ is spanned by the indicator vectors of the weakly connected components of the union of their graph representations (the graph representation of a permutation $\pi \in S_n$ has a vertex for each *i* for $1 \le i \le n$, and a directed edge from *i* to $\pi (i)$ for each *i*). Note that the basis vectors in $B_1$ are orthogonal because their supports are disjoint. We refer to this basis as the *standard basis* of $V_1$.

Likewise, by Eq. (4), a basis $B_2$ for the space $V_2$ can be computed by determining the cycles of $\rho ^{-1} \sigma$ and subtracting the orthogonal projection onto the $\alpha (A,B,C)$ standard basis vectors of $B_1$ from the indicator vector $\chi (C)$ of each cycle *C*. We refer to the resulting basis as the *standard basis* of $V_2$.

The same construction can be applied to $B_3$ and $B_4$, and the overall computation of $B_1$ through $B_4$ takes $O(n^2)$ time. Thus, the most time-consuming step is inverting $B_{14}^{T} B_{14}$ in (11), which requires $O(n^{\omega })$ time, or $O(n^3)$ in practice.

In our running example, with $A' = (13)(28)(57), B' = (16)(27)(34)(58), C' = (12)(34)(56)(78)$, using the notation $e_i$ for the *i*th standard basis and *e* for the vector of all 1’s, we end up with the bases $B_1 = \{e\}$, $B_2 = \{e_2 + e_5 - e/4, e_7 + e_8 - e/4\}$, $B_3 = \{e_1 + e_5 + e_7 - 3e/8, e_3 - e/8, e_4 - e/8\}$, $B_4 = \{0\}$, so by (11),

$$\begin{aligned} M_I = \frac{1}{6} \begin{pmatrix} 4  & {} 2  & {} 0  & {} 0  & {} -2  & {} 2  & {} -2  & {} 2\\ 2  & {} 1  & {} 0  & {} 0  & {} -1  & {} -2  & {} 5  & {} 1\\ 0  & {} 0  & {} 0  & {} 6  & {} 0  & {} 0  & {} 0  & {} 0\\ 0  & {} 0  & {} 6  & {} 0  & {} 0  & {} 0  & {} 0  & {} 0\\ -2  & {} -1  & {} 0  & {} 0  & {} 1  & {} 2  & {} 1  & {} 5\\ 2  & {} -2  & {} 0  & {} 0  & {} 2  & {} 4  & {} 2  & {} -2\\ -2  & {} 5  & {} 0  & {} 0  & {} 1  & {} 2  & {} 1  & {} -1\\ 2  & {} 1  & {} 0  & {} 0  & {} 5  & {} -2  & {} -1  & {} 1\\ \end{pmatrix}. \end{aligned}$$

$M_I$ it is both symmetric, in agreement with Theorem 3, and orthogonal, in agreement with Theorem 4, although it is certainly not genomic. Furthermore, it contains the adjacency (34) common to $B'$ and $C'$, in agreement with Corollary 1. The process of turning it into a genome is the subject of the following section.

## From matrices back to genomes

In this section we describe the two heuristics for extracting back a genome from a symmetric median, in cases when this median is not itself a genomic matrix. The first one is an improvement of the one proposed by Zanetti et al. [6], while the second one is a brute-force approach only applicable in certain cases.

### The first heuristic: maximum-weight matching

Let *M* be a symmetric median to be transformed back into a genome. Since a genome can also be seen as a matching on the extremities of the genes involved, we can construct a weighted graph *H* with a weight of $|M_{ij}| + |M_{ji}| = 2|M_{ij}|$ on the edge from *i* to *j*, provided this weight exceeds $\epsilon = 10^{-6}$, a bound introduced to avoid numerically insignificant values. We modify this by also adding self-loops to *H* with weight $|M_{ii}|$, so that those extremities *i* with a high value of $|M_{ii}|$ can be encouraged to form a telomere. We then extract a maximum-weight matching of *H* by using an implementation of the Blossom algorithm [12]. More specifically, we used the NetworkX package [13] in Python [14], which in turn is based on a detailed paper by Galil [15]. This implementation runs in $O(mn\log n)$ time for a graph with *n* nodes and *m* edges, or in $O(n^3)$ time for dense graphs.

In our running example, the maximum-weight matching is obvious by inspection (in fact, the greedy algorithm yields the optimum matching), and is $M = (34)(27)(58)$. Unfortunately, its score, 10, exceeds the lower bound $\beta = 8$.

### The second heuristic: the closest genome by rank distance

Let *R* be the set of rows of a symmetric, orthogonal median *M* that contain at least one non-integer entry; by symmetry, this is the same as the set of columns that contain at least one non-integer entry. Note that *M* cannot contain a $-1$ value since otherwise, we would have the rest of the row equal to 0 by orthogonality, and its sum would then be $-1$ instead of 1 (as it must be in order to satisfy the lower bound: $A\mathbf {1} = B\mathbf {1} = \mathbf {1}$, so $M\mathbf {1} = \mathbf {1}$ as well, by Corollary 1 in [1]). Hence, *M* must be binary outside of the rows and columns indexed by *R*.

We consider the matrix $M^{R} := M[R,R]$, i.e. the square submatrix of *M* with rows and columns indexed by *R*. We would like to find the genomic matrix *G* closest to $M^{R}$ in rank distance and replace $M^{R}$ with *G* to obtain a candidate genome (since the rest of *M* contains only integers, and *M* is symmetric, any closest genome to all of *M* must necessarily agree with *M* there).

We create an auxiliary graph *H* with a node for each element of *R* and an undirected edge between *i* and *j* if and only if $M^{R}_{ij} \ne 0$. Let $C_1, \ldots , C_k$ denote the connected components of *H*. Our heuristic consists in restricting the search to block-diagonal genomes with blocks determined by $C_1, \ldots , C_k$. Although we did not prove it, we believe that this is in fact sufficient for finding a genomic median. This search can be done in an exhaustive manner if each block has size at most $n = 10$, for which there are only 9496 genomes to test. This can be done quickly—under a second on a modern laptop running R [16]; larger sizes, such as $n = 12$ with over 140,000 genomes to test, take substantially longer.

In our running example, we take $R = [1,2,5,6,7,8]$. There is a single block. We compute that, out of the 76 possible genomes with $n = 6$, only one is at rank distance 1 from $M^{R}$, namely, $M = (14)(25)(36)$, which, after renumbering it according to *R* and adding back the adjacency (34), gives us (16)(27)(34)(58), which happens to be $B'$. It gets a score of 9 with the reduced inputs $A', B', C'$. Although this still exceeds the lower bound $\beta = 8$, an exhaustive check reveals that *M* is one of the three best-scoring genomes, the other two being $M' = (16)(28)(34)(57)$ and $M'' = (16)(25)(34)(78)$. Thus, in this example our second heuristic works better than the first one and, in fact, finds a genomic median.

We conjecture that this happens for any input genomes. In other words, we claim that any genomic median $G^{*}$ of three genomes *A*, *B*, *C* also satisfies

$$\begin{aligned} G^{*} \in \arg \min _{G} r(G-M_I). \end{aligned}$$

We have verified this conjecture for all genomes with $n \le 10$ extremities. We note that while other genomes occasionally attain the minimum rank distance to $M_I$, all the genomes that also attain the smallest possible score *s*(*G*; *A*, *B*, *C*) among genomes are also at a minimum rank distance to $M_I$. If true, our conjecture would potentially provide an alternative way of leveraging the generalized median to search for a genomic median.

### Relationship between the heuristics

We now show that the first heuristic is, in fact, a convex relaxation of the second heuristic. It is common to formulate an approximate search for a matrix *M* of small rank *r*(*M*) by a search for a matrix of small Frobenius norm $||{M||}_{F}$. Here, the Frobenius norm of *M* is the sum of squares of its entries: $||{M||}_{F} = \sum _{i,j} M_{ij}^2$. This is a reasonable approach because the rank of *M* is the number of non-zero entries, sometimes referred to as the $L_0$ norm, of the vector $\mathbf {\sigma } = [\sigma _1, \ldots , \sigma _m]$ of its *singular values*, while the Frobenius norm is the $L_2$ (or Euclidean) norm of the vector $\mathbf {\sigma }$ [17]. The field of compressed sensing [18] frequently uses the approximation of non-convex norms such as the $L_0$ norm by convex ones such as the $L_1$ or $L_2$ norms.

Now, let us consider the problem of finding the genomic matrix *G* that minimizes the Frobenius norm of the difference with a given matrix *M*; the setting here is that *M* is a generalized median of three genomes such as the one found by our algorithm, and *G* is the genomic matrix we want to convert it to. We can write the objective function (more precisely, its square) for this minimization as

$$\begin{aligned} \begin{aligned} f(G) & := ||{M - G||}_{F}^{2} = \sum _{i,j} [M_{ij} - G_{ij}]^2 \\  & = \sum _{i,j} M_{ij}^2 + \sum _{i,j} G_{ij}^2 - 2 \sum _{i,j} M_{ij} G_{ij}. \end{aligned} \end{aligned}$$

However, the term $\sum _{i,j} M_{i,j}^2$ is always constant (in our setting, since *M* is orthogonal, it equals *n*, the number of extremities), and the term $\sum _{i,j} G_{i,j}^2$ is also constant for any genomic matrix *G* (and also equals *n*). Therefore, minimizing *f*(*G*) is equivalent to *maximizing*

$$\begin{aligned} h(G) := \sum _{i,j} M_{ij} G_{ij}, \end{aligned}$$

which is precisely the maximum matching problem applied to *M* because a genome *G* can equivalently be viewed as a matching over the set of *n* extremities.

## Experiments

We tested our algorithm ${\mathcal {A}}$, as well as the two heuristics described in the previous section, on simulated and real data. For our simulations, we started from a random genome with *n* genes, for *n* varying from 12 to 1000, and applied *rn* random rearrangement operations to obtain the three input genomes, with *r* ranging from 0.05 to 0.3, and the rearrangement operations were chosen to be either SCJ (single cut-or-join) [4] or DCJ (double cut-and-join) [19] operations. In both cases the operations are chosen uniformly at random among the possible ones, as described in previous work [6]. For each combination of *n* and *r* we generated 10 samples, for a total of 600 samples for each of SCJ and DCJ.

For the real data, we selected a dataset containing 13 plants from the *Campanulaceæ* family, with the gene order for $n = 210$ gene extremities (i.e. 105 genes) each, and created all possible triples for a total of 286 inputs. We present a summary of our results in the next subsections.

### Results on the SCJ samples

Perhaps because the SCJ rearrangements involve smaller rank distances, the SCJ samples turned out to be particularly easy to process. It turned out that all but 19 (or $\approx 3\%$) of them actually had $\delta = 0$, and all but 5 (or $\approx 1\%$) of them had a median $M_I$ that was genomic. Of these 5 cases, 4 had a submatrix $M^{R}$ of size $n=4$ with all the entries equal to $\pm \frac{1}{2}$, and one had a submatrix $M^{R}$ of size $n=6$ with $\frac{2}{3}$ in each diagonal entry and $\pm \frac{1}{3}$ in each off-diagonal entry.

For those 5 inputs, both the maximum matching as well as the closest genome heuristics resulted in a similar conclusion, namely, that several possible genomes had the exact same distance from $M^{R}$, equal to 1, and all matchings had the same score for the submatrices of size 4. Nevertheless, the solution produced by the maximum matching heuristic (picked arbitrarily among many possible matchings in the case of the submatrices of size 4), namely, the one in which every element of *R* was a telomere, always scored $\beta + 1$ with the original inputs, which was the best possible score among all genomes in every case.

### Results on the DCJ samples

The situation was more complex with the DCJ samples, as 424 out of 600 samples, or more than 70%, had $\delta > 0$, and for 337 out of 600, or more than 56%, $M_I$ had some fractional entries. Unsurprisingly, there was an increasing trend for the proportion of medians $M_I$ with fractional entries as a function of both *n* and *r*. The matching heuristic did not produce very good results, with the score of the resulting genome exceeding the lower bound $\beta$ by a value in the range from 1 to 173, with a mean of 19.

The submatrices $M^{R}$ varied in size from 4 to 354, with a mean size of 64. Nevertheless, over 40% all the fractional cases (135 out of 337) had the largest connected component of size at most 10, so the closest genome heuristic was applicable to them. For those that it was applicable to, the closest genome heuristic produced relatively good results, with the score of the resulting genome exceeding the lower bound $\beta$ by a value in the range from 0 to 21, including one exact match, with a mean of just under 3. It appears that the closest genome heuristic generally exhibits a better performance than the maximum matching heuristic, but is applicable in a smaller number of cases.

### Results on the *Campanulaceæ* dataset

We construct all 286 possible distinct triples of the 13 genomes on $n = 210$ extremities present in our dataset. Out of these, 189 (or 66%) have $\delta = 0$ and 165 (or 58%) have a genomic median $M_I$. For the remaining ones we apply the two heuristics to determine the best one in terms of the score.

The matching heuristic produced reasonable results this time, with deviations from $\beta$ ranging from 1 to 12, and a mean of just over 4. The submatrices $M^{R}$ varied in size from 4 to 22, with a mean size of 9. Nearly two-thirds of them (79/121) had the largest connected component of size at most 10, so the closest genome heuristic was applicable to them. Among those, the deviations from $\beta$ ranged from 1 to 4, with a mean of just over 2. Once again, the closest genome heuristic performed better, but was applicable to a smaller number of cases.

### Running times

The average running time for DCJ samples with $\delta > 0$ of size 100, 300 and 1000, respectively was 0.04, 0.07 and 0.45 s, suggesting a slightly sub-cubic running time; indeed, the best-fitting power law function of the form $f(x) = a x^b$ had $b \approx 2.97$. Both post-processing heuristics were similarly fast to apply, taking an average of 0.5 s for the closest genome and 0.7 s for the maximum matching per instance of the largest size, $n=1000$. The computations were even faster for SCJ samples and real data. By extrapolating these running times, we expect that even much larger instances, with, $n \approx 10^4$, would still run in minutes. We performed all our experiments in the R computing language [16] on a single Mac laptop with a 2.8 GHz Intel Core i7 processor and 16 GB of memory.

## Conclusions

In this work we presented the first polynomial-time exact solution of the median-of-three problem for genomes under the rank distance. Although the resulting median is only guaranteed to be symmetric and orthogonal, not binary, we observed that it frequently happens to be binary (i.e. genomic) with both simulated and real data. For the cases when it is not, we presented two effective heuristics for trying to find the genome closest to the median, and showed that they tend to produce good results in practice.

Despite this important step forward, the fundamental problem of finding the genomic median of three genomic matrices, or, more generally, the permutation median of three permutation matrices, remains open. The additional question of discovering a faster algorithm for the generalized rank median of three genomes (i.e. when there are no restrictions on it being binary) is also open—we conjecture that it is possible to do it in $O(n^2)$.

In future work, we plan to explore the relationships between the rank distance and other well-studied genome rearrangement distances such as the breakpoint distance, DCJ, and SCJ. In addition, we intend to test the suitability of the rank distance for phylogenetic inference, ancestral genome reconstruction, and orthology assignment. Lastly, it would be very interesting to establish the computational complexity of finding the genomic rank median of three genomes.

## Data Availability

The code and the datasets generated and analysed during the current study are available from the corresponding author upon request.

## Appendix

### An example in which all the medians form a multiplicative group

Let $n = 3$ and take the only triplet of distinct genomes for which $\delta (A,B,C) > 0$, namely, $A = (12), B = (13), C = (23)$, i.e. each of the genomes contains a single adjacency as well as a telomere. Note that we identify a permutation with its corresponding matrix in this section. It is easy to see that the identity *I* is a rank median, as are the two 3-cycles $K = (123)$ and $L = (132)$. Using the Maple software [20] we found that all the medians can be written as a subset of the linear combinations of these three “basic” solutions. More precisely,

$$\begin{aligned} {\mathcal {M}} := \{a I + b K + c L | a + b + c = 1  = a^2 + b^2 + c^2 \},  \end{aligned}$$

is the exact description of all the rank medians. It is easy to see (from the properties of the corresponding permutations) that

12 $$\begin{aligned} & I^2 = I^{T} = I, \quad ~L^2 = L^{T} = K, \nonumber \\  & K^2 = K^{T} = L, \quad ~KL = LK = I. \end{aligned}$$

Using these relationships, it is easy to check that the family ${\mathcal {M}}$ is closed under multiplication and inversion—for the latter, it suffices to notice that $(aI + bK + cL)(aI + cK + bL) = I$. Thus, ${\mathcal {M}}$ is a multiplicative group. In fact, it is precisely the set of $3 \times 3$ orthogonal matrices of determinant 1 that fix the vector of all ones, $\mathbf {1}$, or, equivalently, the set of all rotations around the $\mathbf {1}$ axis.

### An example in which not all the medians are orthogonal

Although $M_I$ is always an orthogonal median for three genomic matrices as per Theorems 1 and 4, it is not always true that *every* median of three genomic matrices is orthogonal. We provide a specific counterexample for $n=5$, which was found using the Maple software [20].

Consider the genomes $A = (1\ 3)(2\ 4)$, $B = (1\ 4)(2\ 5)$, and $C = (1\ 2)(3\ 5)$. We have $\beta (A,B,C) = 6$, which is the score that all generalized medians of *A*, *B*, and *C* must obtain. Consider the family of matrices

$$\begin{aligned} M(a) = \begin{pmatrix} a^2 - a  & {} a  & {} 0  & {} 1 - a^2  & {} 0 \\ 1  & {} 0  & {} 0  & {} 0  & {} 0 \\ a - a^2  & {} -a  & {} 1  & {} a^2  & {} 0 \\ a  & {} 1  & {} 0  & {} -a  & {} 0 \\ -a  & {} 0  & {} 0  & {} a  & {} 1 \end{pmatrix}, \end{aligned}$$

where $a \in {\mathbb {R}}$. It is easy to show that $s(M(a);A,B,C) = 6$ for any $a \in {\mathbb {R}}$, so *M*(*a*) is a median of *A*, *B*, and *C*. For $a \ne 0$, however, we see that *M*(*a*) is not orthogonal since, in particular, the second column of *M*(*a*) is not a unit vector.

### Proof of Theorem 2

#### *Proof*

Note that, because of the invertibility of *M*, to prove that *V* is *M*-stable it is sufficient to show that $MV \subseteq V$.

- If *V*, *W* are two *M*-stable subspaces, let $u \in V \cap W$. Then $u \in V$ and $u \in W$, so $Mu \in V$ and $Mu \in W$, and therefore $Mu \in V \cap W$. Hence $M(V \cap W) \subseteq V \cap W$, and $V \cap W$ is *M*-stable.
  Similarly, let $u \in V + W$. Then $u = v + w$ with $v \in V, w \in W$, so $Mu = Mv + Mw \in V + W$, so $M(V+W) \subseteq V + W$, and $V+W$ is *M*-stable.
- Suppose *M* is symmetric and *V* is an *M*-stable subspace. Let $u \in V^{\perp }$, so that $u^{T} v = 0$ for any $v \in V$. Let $w = Mv$; by hypothesis, $w \in V$, so that
  $$\begin{aligned} (Mu)^{T} v=  &  {} u^{T} M^{T} v = u^{T} Mv = u^{T} w = 0, \end{aligned}$$
  since $w \in V$. However, $v \in V$ was chosen arbitrarily, and therefore $Mu \in V^{\perp } \  \forall \  u \in V^{\perp }$, meaning that $M V^{\perp } \subseteq V^{\perp }$, and $V^{\perp }$ is indeed *M*-stable.
- If $M^2 = I = N^2$, let *x* be such that $Mx = Nx$. Then
  $$\begin{aligned} M(Mx) = Ix = x = N(Nx) = N(Mx), \end{aligned}$$
  so that *Mx* is also in the desired subspace $\{x|Mx = Nx\}$, meaning that it is *M*-stable. By symmetry, it is also *N*-stable, completing the proof.

$$\square $$

### Proof that $M_I$ is orthogonal for genomes *A*, *B*, *C*

#### *Proof*

First, we recall that a matrix *M* is orthogonal if and only if

13 $$\begin{aligned} (Mx)^{T} (My) = x^{T}y \quad  \forall \  x,y \in {\mathbb {R}}^n. \end{aligned}$$

Second, it is sufficient to prove that Eq. (13) holds for any pair of vectors in a basis ${\mathbb {B}} = \{v_1, \dots , v_n\}$ of ${\mathbb {R}}^n$. We show this by considering $x = \sum _{i=1}^{n} \alpha _i v_i, y = \sum _{i=1}^{n} \beta _i v_i$, and noting that assuming that (13) holds for the vectors in ${\mathbb {B}}$,

$$\begin{aligned} (M x)^{T} (M y)=  &  {} \sum _{i,j=1}^{n} \alpha _i \beta _j (M v_i)^{T} (M v_j) \\=  &  {} \sum _{i,j=1}^{n} \alpha _i \beta _j v_i^{T} v_j = x^{T} y. \end{aligned}$$

We take ${\mathbb {B}}$ to be the union of the bases for the subspaces $V_i$ for $i=1$ to $i=5$, and consider different cases, once again using the fact that $M_I$ maps vectors in each $V_i$ into other vectors in $V_i$, which follows from Lemma 3 and the fact that $M_I$ fixes each vector in $V_5$. If $x \in V_i, y \in V_j$ with $i \ne j$, without loss of generality $i < j$, then there are three cases to consider.

Case A) $i = 1$ and $j \in \{2, 3, 4, 5\}$; since $V_1$ and $V_j$ are mutually orthogonal, we have $(M_I x)^{T} (M_I y) = 0 = x^{T} y$, since $M_I x \in V_1$ and $M_I y \in V_j$.
Case B) $i \in \{2, 3, 4\}$ and $j = 5$; since $V_i$ and $V_5$ are mutually orthogonal, we have $(M_I x)^{T} (M_I y) = 0 = x^{T} y$, since $M_I x \in V_i$ and $M_I y \in V_5$.
Case C) $i \in \{2, 3\}$ and $j \in \{i+1, \ldots , 4\}$; we consider the case $i = 2$ and $j = 3$, as the others follow by symmetry. Since $M_I = B$ on both $V_2$ as well as $V_3$
  $$\begin{aligned} (M_I x)^{T} (M_I y) =  (B x)^{T} (B y) = x^{T} B^{T} B y = x^{T} I y = x^{T} y. \end{aligned}$$

Now, suppose that *x*, *y* are two vectors from the same subspace, say $x,y \in V_i$. In this case, the matrix $M_I$ acts on $V_i$ via an orthogonal matrix, and the same argument as in the previous equation shows equality, proving the desired result. $\square$

### Proof that $M_I$ is a median for genomes *A*, *B*, *C*

We begin with the following three lemmas, which will be useful in the proof.

#### **Lemma 4**

*If* *V* *is a vector subspace of* ${\mathbb {R}}^n$ *of dimension* *k* *and* *M* *is a square matrix of size* *n*, *then* $MV := \{Mx | x \in V\}$ *is a vector subspace of* ${\mathbb {R}}^n$ *of dimension* $k - d$, *where* $d := \dim (\ker (M) \cap V)$. *Furthermore, for any two subspaces* *V* *and* *W* *of* ${\mathbb {R}}^n$ *and* *M* *a square matrix of size* *n*, $M(V+W) = MV + MW$.

#### *Proof*

The first part of the statement, the fact that *MV* is a vector subspace of ${\mathbb {R}}^n$, is true because

$$\begin{aligned} \alpha _1 M(v_1) + \alpha _2 M(v_2) = M(\alpha _1 v_1 + \alpha _2 v_2), \end{aligned}$$

for any scalars $\alpha _1$ and $\alpha _2$ in ${\mathbb {R}}$ and vectors $v_1$ and $v_2$ in *V*.

The second part can be proven as follows. Let $v_1, \dots , v_d$ be a basis of $\ker (M) \cap V$, and let us extend it to a basis of *V* by adding the vectors $v_{d+1}, \dots , v_k$. Clearly, $Mv_i = 0$ for each $1 \le i \le d$, since $Mx = 0$ for any $x \in \ker (M)$. Furthermore, the $Mv_j$ for $d + 1 \le j \le k$ are linearly independent since

$$\begin{aligned}  &  \sum _{j> d} \alpha _j M v_j = M \left( \sum _{j> d} \alpha _j v_j \right) = 0 \\  & \iff   \sum _{j > d} \alpha _{j} v_{j} \in \ker (M) \cap V \iff \alpha_{j} = 0 \quad  \forall \  j, \end{aligned}$$

where the last conclusion follows from the linear independence of the basis vectors $v_1, \dots , v_k$ and the fact that the first *d* of those form a basis of $\ker (M) \cap V$. Therefore, the space *MV* is spanned by $\{Mv_j\}_{j=d + 1}^{j=k}$, and its dimension is $k - d$.

For the last part, we note that

$$\begin{aligned} x \in M(V+W) & \iff \exists v \in V, w \in W  \text {with} \  x = M(v+w) \\  & \iff \exists v \in V, w \in W  \text {with} \  x = Mv + Mw \\  & \iff x \in MV + MW. \end{aligned}$$

$$\square $$

#### **Lemma 5**

*A* *is an involution on the standard basis* $B_1$ *of* $V_1$ *for genomes* *A*, *B*, *C*.

#### *Proof*

Consider the graph *G* containing the union of the graph representations of the permutations *AB* and *CA*. The standard basis $B_1$ of $V_1$ contains the indicator vectors of the connected components of *G*. We will show that these basis vectors are either fixed or interchanged in pairs by *A*.

By Lemma 3, $A V_1 = V_1$. Now let $C_t$ be a component of *G*, and let $\chi (C_t)$ be its indicator vector. since $\chi (C_t) \in V_1$, the same is true of $\chi (A C_t) := A \chi (C_t)$ by the *A*-stability of $V_1$. However, since *A* is a permutation, $\chi (A C_t)$ is a vector with $|C_t|$ entries equal to 1 and $n - |C_t|$ entries equal to 0. It follows that $A C_t$, the image of the elements of $C_t$ under *A*, is a disjoint union of components of *G*.

Now we show that this disjoint union in fact contains a single component of *G*. Indeed, note that the *A*-stability of $V_1$ means that

14 $$\begin{aligned} & (x_i = x_j \  \forall \  x \in V_1) \iff (x_{\rho (i)} = (Ax)_i = (Ax)_j = x_{\rho (j)} \forall \  x \in V_1). \end{aligned}$$

This shows that whenever *i*, *j* belong to the same component of *G*, then so do $\rho (i), \rho (j)$. Therefore, $A C_t$ must be a single component of *G* for any *t*, and *A* permutes the set of components of *G* by its action, so it is an involution on $B_1$. $\square$

#### **Lemma 6**

*A* *is an involution on the standard basis* $B_2$ *of* $V_2$ *for genomes* *A*, *B*, *C*.

#### *Proof*

Consider the cycles of the permutation *AB*. The standard basis vectors of $V_2$ are the indicator vectors of these cycles, from which we subtract the orthogonal projections onto each of the vectors in $V_1$. We will show that these basis vectors are either fixed or interchanged in pairs by *A*, meaning that *A* is indeed an involution on them.

By Lemma 3, $A V_2 = V_2$. Now let $C_t$ be a cycle of *AB*, and let $\chi (C_t)$ be its indicator vector; the corresponding basis vector of $B_2$ will be given by

15 $$\begin{aligned} v := \chi (C_t) - \sum _{i=1}^{\alpha } \frac{|C_t \cap C_i|}{|C_i|} \chi (C_i), \end{aligned}$$

where the $C_i$ are the components of the graph *G* defining $V_1$. It follows that *Av* is given by

$$\begin{aligned} Av = \chi (A C_t) - \sum _{i=1}^{\alpha } \frac{|C_t \cap C_i|}{|C_i|} \chi (A C_i). \end{aligned}$$

From the proof of Lemma 5, we have $|A C_i| = |C_i| \  \forall \  i$. Furthermore, we have

$$\begin{aligned} |A C_t \cap A C_i| = |A(C_t \cap C_i)| = |C_t \cap C_i|, \end{aligned}$$

since *A* is a permutation. It finally follows that

16 $$\begin{aligned} Av & =  \chi (A C_t) - \sum _{i=1}^{\alpha } \frac{|A C_t \cap A C_i|}{|A C_i|} \chi (A C_i) & = \chi (A C_t) - \sum _{j=1}^{\alpha } \frac{|A C_t \cap C_j|}{|C_j|} \chi (C_j) \end{aligned}$$

where the second equality follows from the fact, shown in the proof of Lemma 5, that *A* permutes the standard basis $B_1$ of $V_1$. Also analogously to the proof of Lemma 5 we can show that $A C_t$ is a single cycle of *AB*. Indeed, it suffices to consider Eq. (14) with $V_1$ replaced by $V_1 + V_2$, which is also *A*-stable.

By combining this fact with Eqs. (15) and (16) we see that the vector *Av* is the basis vector of $B_2$ defined by the single cycle $A C_t$. In fact, $C_t$ and $A C_t$ are either both equally-sized parts of an even cycle in the graph union of the representations of *A* and *B*, or coincide and correspond to a path in that graph. $\square$

#### **Corollary 2**

*Both* *A* *and* *B* *are involutions on the standard basis* $B_2$ *of* $V_2$. *Similarly, both* *B* *and* *C* *are involutions on the standard basis* $B_3$ *of* $V_3$, *and both* *A* *and* *C* *are involutions on the standard basis* $B_4$ *of* $V_4$. *These results also hold for the subspaces* $\ker (A-B) = V_1 + V_2$ *with basis* $B_1 \cup B_2$, $\ker (B-C) = V_1 + V_3$ *with basis* $B_1 \cup B_3$, *and* $\ker (C-A) = V_1 + V_4$ *with basis* $B_1 \cup B_4$.

We will need two additional definitions and three additional simple lemmas.

#### **Definition 4**

Let *A* be a permutation on *n* elements. We denote by *f*(*A*) the number of fixed points of *A*.

#### **Lemma 7**

*Let* *A* *be a permutation on* *n* *elements, let* *f*(*A*) *be as in Definition* 4, *and let* *c*(*A*) *be the number of cycles of* *A*. *Then*

$$\begin{aligned} f(A) \ge 2 c(A) - n, \end{aligned}$$

*with equality if and only if* *A* *is an involution.*

#### *Proof*

The cycles counted by *c*(*A*) can be trivial (fixed points) or non-trivial (size at least 2). There are $c(A) - f(A)$ non-trivial cycles, and they involve $n - f(A)$ elements. It follows that

$$\begin{aligned} & 2(c(A) - f(A)) \le n - f(A) \nonumber \\  & \iff f(A) \ge 2 c(A) - n, \end{aligned}$$

with equality if and only if each non-trivial cycle has size exactly 2, i.e. *A* is an involution. $\square$

#### **Definition 5**

Let *A* and *B* be two involutions. Let *G*(*A*, *B*) be the graph union of the representations of *A* and *B*, which contains paths and even cycles. We define *p*(*AB*) to be the number of paths in *G*(*A*, *B*).

#### **Lemma 8**

*Let* *A* *and* *B* *be two involutions. Then*

$$\begin{aligned} p(AB) = \frac{f(A) + f(B)}{2}. \end{aligned}$$

#### *Proof*

Let *P* be an arbitrary path in *G*(*A*, *B*). Then the endpoints of *P* are two fixed points, one at either end. Since all the fixed points of *A* and *B* form the endpoints of some path, the result follows. $\square$

#### **Lemma 9**

*Let* *A*, *B*, *C* *be three involutions, and let* $\ker (A-B) = V_1 + V_2$ *have the basis* $B_1 \cup B_2$. *Then the number of pairs of distinct basis vectors of* $B_1 \cup B_2$ *that are exchanged by* *A* *(or* *B**) is precisely* $\frac{c(AB) - p(AB)}{2}$.

#### *Proof*

We start by showing that this number is independent of the chosen basis. Note that each pair of vectors (*v*, *w*) that are exchanged by *A* yield an eigenvalue 1 for $v+w$ and an eigenvalue of $-1$ for $v-w$, while any vector *u* that is fixed by *A* yields an eigenvalue 1. Thus, we can diagonalize *A* with respect to any basis on which it is an involution, to get a number of $-1$ eigenvalues equal to the number of exchanged pairs. But the algebraic multiplicity of an eigenvalue is invariant under similarity (similar matrices have the same characteristic equation) [11], so this number, the number of exchanged pairs, is independent of the chosen basis.

Now consider the union graph *G*(*A*, *B*). Each connected component in it is either a path or an even cycle. Each path creates a single cycle in the product *AB* which is fixed by *A* (and *B*). On the other hand, each even cycle splits into a pair of equal-sized cycles in the product *AB*, and those are exchanged by *A* (or *B*). Therefore, if we use the basis of $\ker (A-B)$ consisting of the indicator vectors of the cycles of *AB*, the desired number of pairs is indeed $\frac{c(AB) - p(AB)}{2}$. $\square$

We are now ready to prove our main result. We begin by proving it for the case $\alpha = 1$, and then generalize it to arbitrary $\alpha$.

#### **Theorem 6**

*The matrix* $M_I$ *is a median of genomes* *A*, *B*, *C* *if* $\alpha (A,B,C) = 1$.

Let us first define $V_{12}$ to be the restriction of $V_1 + V_2$ to those vectors which are fixed by *A* (equivalently, *B*). In other words, let $V_{12} := (V_1 + V_2) \cap \ker (A-I)$.

We begin with the decomposition of ${\mathbb {R}}^n$ from Zanetti et al. [6], to which we apply $(C-I)$:

17 $$\begin{aligned} {\mathbb {R}}^n & = V_1 + V_3 + V_1 + V_4 + V_1 + V_2 + V_5 \nonumber \\  & \supseteq (V_1 + V_3) + (V_1 + V_4)  + (V_{12} + V_5); \nonumber \\ (C-I) {\mathbb {R}}^n & \supseteq (C-I) (V_1 + V_3) + (C-I)(V_1 + V_4)  + (C-I)(V_{12} + V_5). \end{aligned}$$

We will show that the sum on the right-hand side of Eq. (17) is direct. We will then compute the dimension of each term to reach the desired conclusion.

First, we show that $(C-I) (V_1 + V_3)$ and $(C-I) (V_1 + V_4)$ are disjoint subspaces, so that the sum of the first two terms is direct.

#### **Lemma 10**

$$\begin{aligned} & (C-I) (V_1 + V_3) \cap (C-I) (V_1 + V_4) = \{0\}. \end{aligned}$$

#### *Proof*

We reason as follows.

$$\begin{aligned} x & \in (C-I) (V_1 + V_3)  \cap (C-I) (V_1 + V_4) \\  & \iff \exists v \in \ker (B-C),  w \in \ker (C-A)  \text {s.t.} \  (C-I)v = x = (C-I)w \\  & \iff (B-I)v = x = (A-I)w. \end{aligned}$$

Now, by Lemma 3, $(B-I) \ker (B-C) \subseteq B \ker (B-C) - \ker (B-C) \subseteq \ker (B-C)$ by the *B*-stability of $\ker (B-C)$, and similarly, $(A-I) \ker (C-A) \subseteq \ker (C-A)$ by the *A*-stability of $\ker (C-A)$. Since *x* is in their intersection, we get $x \in V_1$.

However, since $\mathbf {1}^{T}x = \mathbf {1}^{T}(B-I)v = 0^{T} v = 0$, it follows that $x = 0$ because when $\alpha = 1$, $V_1$ is spanned by $\mathbf {1}$, meaning that the subspaces are indeed disjoint. $\square$

We now show that the addition of the third term in Eq. (17) keeps the sum direct.

By the same reasoning as in the proof of Lemma 10, we see that $C - I$ maps both $V_1 + V_3 = \ker (B-C)$ and $V_1 + V_4 = \ker (C-A)$ into themselves.

Since $V_1 + V_2 = \ker (A-B)$, we get

$$\begin{aligned} V_{12} \subseteq \ker (A-B) \cap \ker (A-I) = \ker (A-I) \cap \ker (B-I). \end{aligned}$$

We will now show that $(C-I)V_{12} \subseteq {{\,\text{im}\,}}(C-A)$. Indeed, we have

$$\begin{aligned}  &  y \in (C-I)V_{12} \implies y = (C-I)x, x \in \ker (A-I)  \cap \ker (B-I)\\  & \quad \implies Ax = x = Bx\\  &  \quad \implies y = Cx - x = CAx - AAx  = (C-A)Ax \in {{\,\text{im}\,}}(C-A). \end{aligned}$$

By the same reasoning, $(C-I)V_{12} \subseteq {{\,\text{im}\,}}(B-C)$.

Furthermore, we have $V_5 \subseteq {{\,\text{im}\,}}(B-C) \cap {{\,\text{im}\,}}(C-A)$, and both ${{\,\text{im}\,}}(B-C) = \ker (B-C)^{\perp }$ as well as ${{\,\text{im}\,}}(C-A) = \ker (C-A)^{\perp }$ are *C*-stable by parts b and c of Theorem 2, and their intersection is also *C*-stable by part a of this theorem. It follows that

$$\begin{aligned} (C-I)V_5\subseteq  CV_5 - V_5 \subseteq  {{\,\text{im}\,}}(B-C) \cap {{\,\text{im}\,}}(C-A). \end{aligned}$$

By combining this with the previous results on $(C-I)V_{12}$, we conclude that

$$\begin{aligned}  (C-I)(V_{12} + V_5) & \subseteq (C-I)V_{12} + (C-I)V_5 \\  & \subseteq {{\,\text{im}\,}}(B-C) \cap {{\,\text{im}\,}}(C-A). \end{aligned}$$

Since ${{\,\text{im}\,}}(B-C) \cap {{\,\text{im}\,}}(C-A)$ is orthogonal to the sum of $V_1 + V_3$ and $V_1 + V_4$, which equals $\ker (B-C) + \ker (C-A)$, it follows *a fortiori* that $(C-I)(V_{12} + V_5)$ is disjoint from the sum of these subspaces, so the sum in Eq. (17) is direct.

We now consider the dimension of each of the terms in Eq. (17).

Since *C* permutes the basis vectors of $V_1$, $V_3$ and $V_4$ by Lemmas 5 and 6, the dimension of $\ker (C-I) \cap (V_1 + V_3)$ equals the number of those basis vectors that *C* maps into themselves, plus the number of pairs of basis vectors that get swapped by *C*. It follows by Lemmas 4 and 9 that

$$\begin{aligned} & \dim ((C-I)(V_1 + V_3)) \\  & \quad = \dim (V_1 + V_3) - \dim (\ker (C-I) \cap (V_1 + V_3)) \\  & \quad = c(BC) - p(BC) - \frac{c(BC)-p(BC)}{2} \\  & \quad = \frac{c(BC)-p(BC)}{2}. \end{aligned}$$

In the same way, we get

$$\begin{aligned} \dim ((C-I)(V_1 + V_4)) = \frac{c(CA)-p(CA)}{2}. \end{aligned}$$

Analogously, by using Lemmas 4 and 9 once again, we have

$$\begin{aligned} \dim (V_{12}) & =  \dim ((V_1 + V_2) \cap \ker (A-I))\\  & = \dim (V_1 + V_2) - \dim ((A-I)(V_1 + V_2))\\  & = n_2 + \alpha (A,B,C) - \frac{c(AB)-p(AB)}{2}, \end{aligned}$$

where $$n_2 := \dim (V_2)$$.

Lastly, by Lemma 4 the dimension of ${{\,\text{im}\,}}(C-I) = (C-I) {\mathbb {R}}^n$ equals

$$\begin{aligned} & \dim ({\mathbb {R}}^n) - \dim (\ker (C-I) \cap {\mathbb {R}}^n) = n - c(C). \end{aligned}$$

From the directness of the sum in the second part of Eq. (17), we have

$$\begin{aligned} n - c(C)\ge  &  {} \dim ((C-I) (V_{12} + V_5)) \\  & + \dim ((C-I)(V_1 + V_3))\\  & + \dim ((C-I)(V_1 + V_4)) \\=  &  {} \dim ((C-I) (V_{12} + V_5)) \\  & + \frac{c(BC)-p(BC)}{2} + \frac{c(CA)-p(CA)}{2} \end{aligned}$$

which implies

$$\begin{aligned} & \dim ((C-I) (V_{12} + V_5)) \\  & \quad \le n - c(C) - \frac{c(BC)-p(BC)}{2} - \frac{c(CA)-p(CA)}{2}. \end{aligned}$$

By using Lemmas 7 and 8, the definition of $n_2,$ and the invariants $\alpha (A,B,C)$, $\beta (A,B,C)$, and $\delta (A,B,C)$ we can rewrite the right-hand side above to obtain

$$\begin{aligned} & \dim ((C-I) (V_{12} + V_5)) \\  & \quad \le n - c(C) - \frac{c(BC) + c(CA)}{2}  + \frac{p(BC) + p(CA)}{2}\\  & \quad = n - c(C) - \frac{c(AB) + c(BC) + c(CA)}{2}  + \frac{c(AB)}{2} + \frac{f(A) + f(B)}{4} + \frac{2c(C) - n}{2} \\  & \quad = \frac{n + c(AB)}{2} - \frac{3n - 2 \beta (A,B,C)}{2}  + \frac{f(A) + f(B)}{4}\\  & \quad = \beta (A,B,C) - n + \frac{c(AB)}{2} + \frac{p(AB)}{2} \\  & \quad = c(AB) - \frac{c(AB) - p(AB)}{2} + \beta (A,B,C) - n \\  & \quad = n_2 + \alpha (A,B,C) + \beta (A,B,C) - n  - \frac{c(AB) - p(AB)}{2} \\  & \quad = n_2 + \delta - \frac{c(AB) - p(AB)}{2}. \end{aligned}$$

And now we use Lemma 4 and the fact that $\dim (V_5) = 2 \delta$ to obtain

$$\begin{aligned} \dim (V_I^{C}) &=   \dim (\{x + y | x \in V_2, y \in V_5, \\  & \quad C(x+y) = Ax + y\}) \\ & \ge \dim (\{x + y | x \in V_{12}, y \in V_5, \\  & \quad C(x+y) = Ax + y\}) - 1\\ &=   \dim (\{x + y | x \in V_{12}, y \in V_5, \\  & \quad C(x+y) = x+y\}) - 1\\ &=   \dim (\ker (C-I) \cap (V_{12} + V_5)) - 1\\ & =   \dim (V_{12} + V_5) - \dim ((C-I) (V_{12} + V_5)) \\ & \quad - 1 \ge  n_2 + \alpha (A,B,C)  - \frac{c(AB)-p(AB)}{2} \\ & \quad + 2 \delta - \biggl (n_2 + \delta - \frac{c(AB) - p(AB)}{2} \biggr ) - 1 \\   & =  \delta . \end{aligned}$$

Therefore, all the intermediate inequalities are equalities as well. This proves that $M_I$ is always a median for three involutions provided $\alpha (A,B,C) = 1$. Note that we subtract 1 in the first step above to account for the fact that any multiple of the vector $\mathbf {1}$ can be added to any solution of the set of equations defining $V_I^{C}$.

### Proof that $M_I$ is a median for general $\alpha$

This time we use a slightly different decomposition of ${\mathbb {R}}^n$ because the intersection of $(C-I)(V_1 + V_3)$ and $(C-I)(V_1 + V_4)$ may be non-trivial. Namely, we replace Eq. (17) with

18 $$\begin{aligned} & (C-I) {\mathbb {R}}^n\supseteq  (C-I) V_1 + (C-I) V_3  \\ & \quad + (C-I) V_4 + (C-I)  (V_{12} + V_5). \end{aligned}$$

We will show that the resulting sum is direct.

First, we note that, because of the *C*-stability of $V_1, V_3$, $V_1 + V_3$, and $V_4$, we have that $(C-I) V_1 \cap (C-I) V_3 \subseteq V_1 \cap V_3 = \{0\}$, and furthermore, $$ \begin{aligned} & ((C-I) V_1 + (C-I) V_3) \cap (C-I) V_4 \\ & = (C-I) (V_1 + V_3) \cap (C-I) V_4 \subseteq (V_1 + V_3) \cap V_4 = \{0\}\end{aligned}$$, where we used the last part of Lemma 4 in the second step.

Second, by the last part of Lemma 4, we have that $$\begin{aligned} (C-I) V_1 + (C-I) V_3 + (C-I) V_4 & = ((C-I) V_1 + (C-I) V_3) \\ & \quad + ((C-I) V_1 + (C-I) V_4) = (C-I) (V_1 + V_3) + (C-I) (V_1 + V_4)\end{aligned}$$.

We already showed in the previous section that the intersection of the sum $(C-I) (V_1 + V_3) + (C-I) (V_1 + V_4)$ with $(C-I)(V_{12} + V_5)$ is trivial. It follows that the sum in Eq. (18) is indeed direct.

Now we consider the dimension of each term. Let us define *q* as the dimension of $(C-I)V_1$ (it is not simple to express in terms of other basic quantities, but we will see that it cancels out at the end). By the directness of the sum in Eq. (18), and reasoning in the same way we did in the previous section, we have

$$\begin{aligned} & \dim ((C-I)V_1) + \dim ((C-I)V_3) \\  & \quad = \dim ((C-I)V_1 + (C-I)V_3) = \dim ((C-I) (V_1 + V_3)) = \frac{c(BC)-p(BC)}{2}, \end{aligned}$$

and similarly,

$$\begin{aligned} & \dim ((C-I)V_1) + \dim ((C-I)V_4) \\  & \quad = \dim ((C-I) (V_1 + V_4)) \\  & \quad = \frac{c(CA)-p(CA)}{2}. \end{aligned}$$

Therefore

$$\begin{aligned} & \dim ((C-I)V_1 + (C-I)V_3 + (C-I)V_4) \\  & \quad = \frac{c(BC)-p(BC)}{2} + \frac{c(CA)-p(CA)}{2} - q. \end{aligned}$$

By repeating the calculation in the previous subsection, but carrying the extra *q* term throughout, we now obtain the upper bound

$$\begin{aligned} & \dim ((C-I) (V_{12} + V_5)) \\  & \quad \le n_2 + \delta - \frac{c(AB) - p(AB)}{2} + q. \end{aligned}$$

And now, we have to carefully estimate the number of degrees of freedom gained by going from $V_I^{C} := \{x + y | x \in V_2, y \in V_5, C(x+y) = Ax + y\}$ to the potentially larger subspace $\{x + y | x \in V_{12}, y \in V_5, C(x+y) = Ax + y\}$ (this was simple in the previous section since there was at most 1 extra dimension when $\dim (V_1) = \alpha = 1$).

We first restrict the space $V_I^{C}$ to allow only those vectors *x* for which $Ax = x$, i.e. we replace it with

19 $$\begin{aligned} & \{x + y | x \in V_2 \cap \ker (A-I), y \in V_5, C(x + y) = Ax + y\}. \end{aligned}$$

This restriction clearly does not increase its dimension.

Second, we go from this subspace to the subspace

20 $$\begin{aligned} & \{x + y | x \in V_{12}, y \in V_5,  C(x+y) = Ax + y\}. \end{aligned}$$

Recall that $V_{12} := (V_1 + V_2) \cap \ker (A-I)$. By Lemmas 5 and 6, *A* is an involution on the standard bases of both $V_1$ and $V_2$, and these bases can be altered so that each pair of basis vectors *v* and *w* permuted by *A* is replaced by $v + w$ and $v - w$, of which the first one is in $\ker (A-I)$ and the second one is not. Together with the vectors *u* fixed by *A*, which are also in $\ker (A-I)$, the resulting bases will contain sub-bases for the intersection of the corresponding vector space with $\ker (A-I)$. It follows that $V_{12} = (V_1 \cap \ker (A-I)) + (V_2 \cap \ker (A-I))$.

We note that in general, for three finite-dimensional vector spaces *U*, *V*, *W*, we have $(U \cap W) + (V \cap W) \subseteq (U + V) \cap W$, and the inclusion can be strict; however, we have equality here thanks to the representation of *A* on $V_1 + V_2$.

It is now easy to see from the foregoing discussion that the subspace in Eq. (20) differs from the one in Eq. (19) by the vectors in the subspace

$$\begin{aligned} & V_1 \cap \ker (A-I) \\  & \quad = \{x \in {\mathbb {R}}^n| x = Ax = Bx = Cx\} \\  & \quad = V_1 \cap \ker (C-I), \end{aligned}$$

whose dimension, by Lemma 4, is given by

$$\begin{aligned} & \dim (V_1 \cap \ker (C-I)) \\  & \quad = \dim (V_1) - \dim ((C-I) V_1) \\  & \quad = \alpha - q. \end{aligned}$$

The final calculation from the previous section (with some parallel intermediate steps omitted) now becomes

$$\begin{aligned} & \dim (V_I^{C}) = \dim (\{x + y | x \in V_2, y \in V_5, \\  & \qquad \quad C(x+y) = Ax + y\}) \\  & \quad \ge \dim (\{x + y | x \in V_2 \cap \ker (A-I), \\  & \qquad \quad y \in V_5, C(x + y) = Ax + y\}) \\  & \quad \ge \dim (\{x + y | x \in V_{12}, y \in V_5, \\  & \qquad \quad C(x+y) = Ax + y\}) - (\alpha - q)\\  & \quad \ge n_2 + \alpha - \frac{c(AB)-p(AB)}{2} + 2 \delta \\  & \qquad - \biggl (n_2 + \delta - \frac{c(AB) - p(AB)}{2} + q \biggr ) \\  & \qquad - (\alpha - q)\\  & \quad = \delta , \end{aligned}$$

which completes the proof.

## Abbreviations

DCJ: double-cut-and-join
SCJ: single cut-or-join
NP: non-deterministic polynomial time
